# Effective Microorganisms and Glass Nanopowders from Waste Bottle Inclusion on Early Strength and Microstructure Properties of High-Volume Fly-Ash-Based Concrete

**DOI:** 10.3390/biomimetics7040190

**Published:** 2022-11-04

**Authors:** Ali M. Onaizi, Ghasan Fahim Huseien, Nor Hasanah A. Shukor Lim, W. C. Tang, Mohammad Alhassan, Mostafa Samadi

**Affiliations:** 1 Institute for Smart Infrastructure and Innovative Construction, School of Civil Engineering, Faculty of Engineering, Universiti Teknologi Malaysia, Pagoh 81310, Malaysia; 2 Institute of Architecture and Construction, South Ural State University, Lenin Prospect 76, 454080 Chelyabinsk, Russia; 3 School of Architecture and Built Environment, The University of Newcastle, University Drive, Callaghan, NSW 2308, Australia; 4 Civil Engineering Department, Jordan University of Science and Technology (JUST), Irbid 22110, Jordan; 5 Civil Engineering Department, Al Ain University (AAU), Al Ain 64141, United Arab Emirates

**Keywords:** effective microorganisms, wastes glass, fly ash, early strength, microstructures

## Abstract

In concrete production, the use of high-volume fly ash (FA) as the cement substitute became interesting to achieve more sustainable and eco-friendly construction materials. However, concrete produced using high volumes of FA as cement substitute suffers from various limitations such as low strength at early ages. Considering the engineering solutions and economy of FA-included concrete, it has become vital to address such issues. In this perception, some concrete mixes were designed using more abundant and low-cost local waste materials such as waste glass bottle nanopowders (WGBNPs) and effective microorganisms (EMs) to determine the feasibility of compensating for the strength loss at early ages due to FA inclusion. The proposed mixes contained 10% of EMs as water replacement, 50% of FA, and various percentages of WGBNPs as cement replacement. The effects of EMs and WGBNPs inclusion on the early strength and microstructure properties of the produced FA-based concrete mixes were determined. The results show that the strength indexes of the concrete at all test ages were improved due to WGBNP and EM incorporation. At almost all curing ages, the mechanical performance of the concrete made with 10% EMs and 4% WGBNPs was comparable to that of normal concrete (control mix), wherein the mix containing 6% WGBNPs outperformed the control mix. The microstructure analysis of the studied mixes revealed an increase in the hydration products, structural compactness, and homogeneity due to the synergy of WGBNPs and EMs, especially the specimen made using 10% EMs and 6% WGBNPs. It is established that the proper utilization of EMs and WGBNPs in FA-based concrete can be beneficial for waste recycling and landfill problems, thus lowering environment pollution.

## 1. Introduction

The adoption of various industrial by-products (as wastes) in the concrete industry seems to be environmentally and economically beneficial due to their abundance, low carbon footprint, and affordability. This is urgently needed because of the ever-growing environmental concern related to cement uses and various issues concerning landfills because of waste from different industries [1,2]. The effective use of FA in concrete can be a pioneering move to develop more sustainable and highly durable construction materials [3,4]. Even though FA has been a hot topic for extensive research over the past decade, experts have found some contradictory findings regarding the mechanical and durability properties of high-volume fly ash (FA)-based concrete. In addition, most of the previous studies revealed that FA can enhance workability, reduce hydration heat and thermal cracking in the cementitious composites, and improve mechanical and durability properties, primarily at late ages [5,6]. However, due to the slower pozzolanic reaction of FA than OPC, high volumes of FA-based concrete exhibit a marked decline in strength properties at early ages [3]. Despite a decrease in the strength at early ages, the strength gain in FA-based concrete is maintained for a significantly longer period of time compared with normal concrete [7]. This indicates that FA requires an extended curing period to hydrate, which causes a delay in the pursuance for the construction applications. In short, overcoming the declining tendency of the strength at early ages remains challenging. Despite many efforts, no precise solutions have yet been achieved with clear standards and limitations.

Numerous studies have addressed different solutions to surmount the decrease in the early ages strength properties of FA-based concretes, including an increase in the fineness of FA particles [8,9,10], increase in the alkalinity of concrete mixes by the addition of more sodium hydroxide (Ca(OH)_2_) [11,12], use of nanostructures [13], inclusion of EMs [14], and so forth. Among all these strategies, the incorporation of nanomaterials generated renewed interests because of their outstanding ability to repair flaws of concrete at early stages, particularly the mixes containing high volumes of FA. Li Gengying [15] used nano-SiO_2_ to improve the early strength of high-volume FA-based concrete and, compared with that made without nano-SiO_2,_ as early as after 3 days curing, improvements in the pore size distribution of the suggested concrete were observed. Shaikh and Supit [16] reported that the inclusion of calcium carbonate (CaCO_3_) nanoparticles in high-volume FA concrete mixtures significantly improve both early age and later age compressive strength. However, most of the nanomaterials that have been introduced as effective solutions are either rare or too expensive to prepare [17]. Therefore, the utilization of various local waste materials such as WGBNPs and EMs seems to be the most effective solution. The presence of noncrystalline alumina and silica in the pulverized glass from waste bottles make them very efficient pozzolanic materials [18,19].

According to ASTM C618-19 [20], the glass powders from bottles can be classified as pozzolanic material due to the presence of a high amount of calcium, aluminum, and silicon in the amorphous phase. Several studies have recently been conducted to determine the feasibility of using waste glass as aggregates in concrete [21,22,23]. Nevertheless, the results reveal that concrete mixes composed of waste bottle glass particles as coarse or fine aggregates perform poorly because of strong alkali and silica reactions related damages [24,25,26]. Furthermore, with the reduction in the fineness of glass aggregates below 20 μm, the resultant glass powders exhibit favorable pozzolanic attributes with weak alkali and silica reaction [18,19]. Zhang et al. [27] investigated the impact of various particle sizes of glass powders on the mechanical and microstructure properties of concrete. The particle size distribution of glass powders was found to have a significant effect on the overall properties of concrete. An increase in the fineness of glass powders could enhance pozzolanic reactivity due to a chemical reaction with Ca(OH)_2_ to form extra calcium silicate hydrate gels by avoiding alkali and silica reactions [19]. This occurrence generated new prospects to use glass powders in the manufacturing of concrete mixes as a partial replacement for OPC.

Many investigations [28,29,30,31,32,33] displayed sufficient pozzolanic properties of glass powder and its use as partial replacement for cement, becoming advantageous for improving the overall bulk properties of concrete. Sancheti et al. [34], Elmoaty et al. [32], and Rahman et al. [30] investigated the possibility of implementing GPs as a partial replacement for cement at different contents in the range of 5–25% of the binder weight. The results display an enhancement of the compressive strengths (CS), flexural strengths (FS), water absorptions (WA), and chloride ions penetration, especially the mix made using 15% glass powder as cement substitute. Yet, all these reports showed a reduction in the strength properties of concrete made with high substitutions of glass particles. Elaqra et al. [35] and Deng et al. [36] used fine powders of glass in different mixes without and with the addition of coal ash. An enhancement in the CS, FS, splitting tensile strength (STS), bending toughness, and deflection was recorded with the addition of glass powder as substitute in the range of 10–20% of the binder weight. Xi et al. [37] studied the effect of waste glass powder with a median size of 18.21 μm as a supplementary cementitious material on the physical and mechanical properties of cement paste. The results reveal that glass powder has remarkable pozzolanic reactivity, wherein the dissolved silica from glass can strongly react with Ca(OH)_2_ and participate in the production of C–S–H gels. However, all concrete specimens prepared with glass powder showed lower strength performance, where the lower rate in the strength ranged from 1.0–4.2% compared with the one prepared without glass powders [37].

Clearly, most of the aforementioned previous research has focused on using glass powders as cement substitutes. However, the research related to the utilization of glass as nanomaterial additive to improve the early strength properties of high-volume FA-based concrete is not matured yet. Huseien et al. [38] demonstrated that the addition of WGBNPs in the geopolymer concrete has a positive effect on the strength properties and water absorption. The observed improvement in the strength and microstructure properties of WGBNPs-included concrete in various studies [19,36,38,39] was ascribed to the pozzolanic reaction mechanism and the creation of additional hydration gels and physical fillings of pores, voids, and cracks, significantly influencing the homogeneity, density, and strength characteristics of the cementitious products.

Lately, EMs have emerged as innovative bio-concrete components in the concrete industry [40,41]. They are abundant in soil, oil, and water reservoirs, as well as wastewater effluents from various industries [42]. Diverse approaches can be utilized to integrate EMs into bio-concrete [43]. One of these methods involves the integration of microbial broth directly into the fresh concrete as a partial replacement for water [44,45]. EMs have shown immense benefits when incorporated into concrete [44], wherein the strength properties of the fresh mixes (particularly at the early ages) and cement hydration reaction processes were improved, thus enhancing the microstructures [44,46]. In addition, EMs have significant influence on the viscosity of the prepared mixing water (Ems–Water) [45]. Hussein et al. [42] reported an increase in the viscosity of Ems–Water from 0.95 to 1.25 mPa (as the mixing water replacement) when the EMs content was increased from 0 to 25% [44]. Wang et al. [47] claimed that added water viscosity has strong potential to influence both fresh and hardened properties of concrete. This increase in viscosity can reduce the bleeding, segregation, and settlement, facilitating the production of liquid concrete with high consistency. In addition, the lowering surface tension forces of the mixing solution can catalyze the cement component to react faster, enhancing the durability performance, hydration process, and strength properties of concrete at earlier ages [48]. However, the studies concerning the effect of EMs inclusion into high-volume FA-based concrete is still lacking.

A recent study [44] aimed to determine the optimum replacement percentage of mixing water with EMs (5–25 wt.% of mixing water). The results reveal that inclusion of 10% of EMs as mixing water replacement into high-volume FA-based concrete (containing 50% of FA) achieved a satisfactory strength performance. An enhancement in the strength by 42.6 and 14.5% was observed at 3 and 28 days, respectively, which was ascribed to the better filling of pores through C-A-S-H gel due to reduced water surface tension and acceleration of the hydration process [45]. As a result, the pore size and pore ratio was minimized, enabling the development of high-density microstructures [49]. However, the strength of the EMs-added concrete was decreased by 4.5 and 23.7% at 3 and 28 days, respectively, compared with the control sample [44]. A further drop in pH was observed due to a rise in EMs that in turn lowered the alkalinity of the pore solution and eliminated the positive effects achieved by the optimum EMs. It was noted that the reduction in the fluid alkalinity and pores could have a negative impact on the hydration reaction, resulting in a lower strength of the concrete at early ages [50]. Additionally, a low pH can delay the FA hydration, thus preventing microstructure development in high-volume FA-based concrete [51].

In this view, the present work aimed to make some sustainable concrete mixes for diverse applications for the construction purposes with reduced ecological issues such as carbon dioxide release, energy utilization, and landfill dumping through the replacement of OPC by high volumes of FA (50%) integrated with WGBNPs and EMs. The WGBNPs and EMs were utilized to resolve the problems related to early strength traits and improve the mechanical and microstructural characteristics at both early and late ages. Diverse tests such as CS, FS, TS, WA and X-ray diffraction (XRD), scanning electron microscopy (SEM), energy-dispersive X-ray spectroscopy (EDS), and thermogravimetric analysis (TGA) were carried out to determine various properties of the proposed concrete mixes.

## 2. Material Design, Proportion, and Methods

### 2.1. Material Design

**Figure 1:**
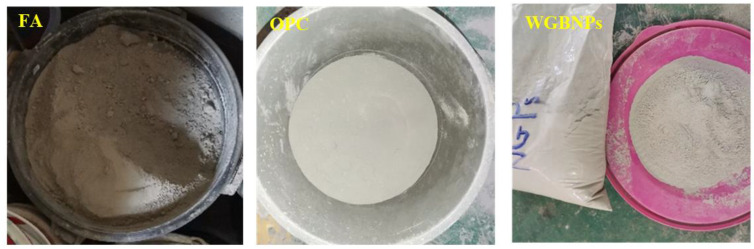
Raw material-based concrete binder.

The modified concrete was synthesized using OPC, FA, Ems, and WGBNPs (Figure 1). Herein, OPC fulfilled the ASTM C150 requirement [52] for cement Type I that was obtained from a local cement factory in Malaysia and utilized as the main resource of calcium oxide (CaO). FA was obtained as the main source of aluminum silicate from the Tanjung Bin power station (Johor, Malaysia) and utilized as it is (no treatment), apart from grinding the larger particles. Waste glass bottles were first acquired from Skudai (Malaysia), followed by cleaning using normal tap water to eradicate the contaminants and then ground by a crusher machine (Los Angeles abrasion machine of capacity 25 kg, Skudai, Johor, Malaysia) for 4 h to obtain a medium particle size of 25 µm. Again, the powder was crushed for another 7–8 h in a ball mill machine to ensure the best distribution and homogeneity of nanoparticles.

**Figure 2:**
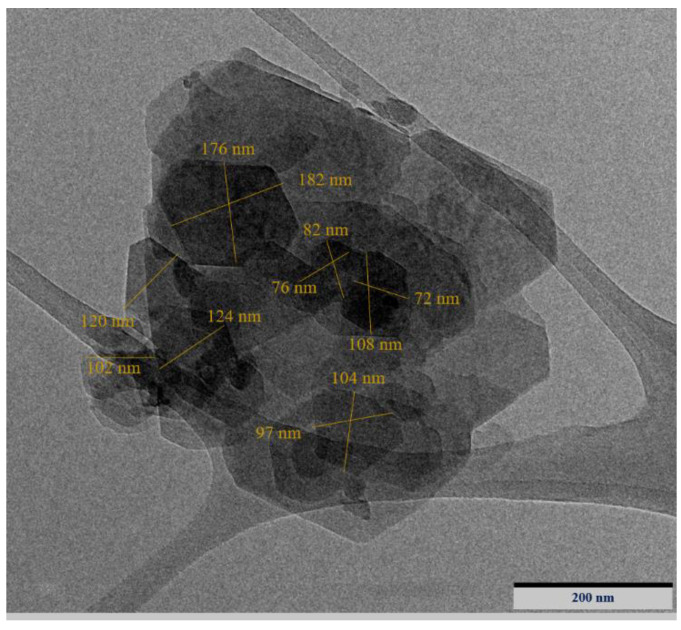
TEM image of WGBNPs.

X-ray fluorescence spectroscopy (XRF, Tokyo, Japan) was used to study the chemical compositions of OPC, FA, and NGBWP (Table 1). The quantity of Ca, Al_2_O_3,_ and SiO_2_ were 89.97% in OPC, 91.17% in FA, and 86.16% in WGBNPs. OPC enclosed a significant quantity of CaO (about 67.84%), while FA contained a relatively small amount (5.16%). The amount of K_2_O was below 1% in OPC, FA, and WGBNP. Meanwhile, the amount of Fe_2_O_3_ was almost the same as OPC (3.35%) and FA (3.67%). Generally, the XRF results indicate that OPC contained a high amount of Ca, and FA and WGBNP enclosed a high amount of aluminum and silicon, respectively. The physical properties of OPC, FA, and WGBNP, such as specific gravity, color, and mean size, are shown in Table 1. The specific gravities of OPC, FA, and WGBNP were, correspondingly, 3.15, 2.20, and 1.02. Furthermore, the appearance of OPC was dark gray, FA was gray, and WGBNP was light gray. The TEM image of WGBNPs is illustrated in Figure 2, and the estimated average particle size of WGBNP was around 112 nm.

**Figure 3:**
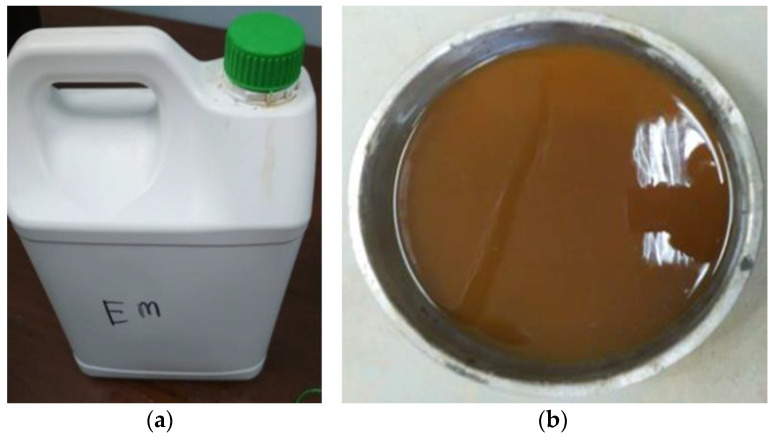
Ems solution (**a**) EMs-1 (**b**) EM substituted with 10% water.

The EMs were generated via the usual fermentation process, not genetically engineered or chemically metabolized. The EMs are a mixture made of various advantageous microorganisms that arise naturally or are enclosed in foods. These EMs are not genetically modified organisms. In addition, they are not pathogenic, harmful or chemically synthesized. When EMs are exposed to the natural atmosphere, the influences of individual microorganisms are very much evident. In this study, the EMs of type EMs-1, weighing 5 kg (Figure 3a), were obtained in a sealed container from Peladang (Johor Bahru, Malaysia). To activate the EMs-1, 5% was introduced into a solution composed of 90% water and 5% blackstrap molasses served as the main food resource, providing the EM-activated solution (EMs-AS). The EM solution included *Bacillus subtilis* and *lactic acid* bacteria. Generally, these bacteria are considered harmless without any negative effect on humans or environments. In addition, EMs can improve the activity of EMs-AS. To activate these bacteria, a pH of less than 4 was achieved by fermenting them without oxygen supply for about 7 to 10 days. The bacteria were utilized without any protection. The physical characteristics and solubility of water, EMs-AS, and water with 10% EMs were observed to visualize the fresh and solid concrete colors. Consequently, EMs were initially dark brown in color, but the EM solution became dark yellowish after being substituted with 10% water (Figure 3b). Several chemical and physical tests (Table 2) were performed at the Environment Laboratory of Universiti Teknologi Malaysia to further characterize the studied EMs. The primary idea of adding EM in concrete is to make a novel concrete mix that uses living organisms with improved characteristics. An understanding of the bacterial activity towards cement hydration, various chemical phases, and microstructure examination enabled us to further look at the applications of microorganisms in concretes. These bacteria in concrete could generate an enzyme called urease that catalyzes urea (CO(NH_2_)_2_) into ammonium ions (NH_4_^+^) and carbonate radicals (CO_3_^2−^).

Table 2 indicates the effects of Ems–water replacement on the characteristics of the produced solution. The EMs and tap water had pH values of 3.5 and 6.7, respectively. As the amount of EMs in the solution increased from 0 to 10%, the pH of the solution became more acidic, changing from 6.7 to 6.3.

The XRF study was performed to determine the elemental composition by the mass proportion of EMs. Mg (0.1140%), Al (0.0316%), Si (0.0546%), P (0.0233%), K (0.7670%), Ca (0.981%), Zr (0.0148%), Ru (0.214%), Rh (97.5%), and Pr (97.5%) were found to be the most abundant elements in the EMs (0.0125%). Rhodium had the highest percentage by mass in the EMs (97.5%), which is most commonly found in river sand or agricultural soil. In addition, the soil included various beneficial microorganisms, wherein Rhodium can also be used in the corrosion protection.

In this experiment, natural siliceous river sand was used as fine aggregates. First, the collected sand was cleaned following the ASTM C117 to remove silts and impurities. Next, it was dried in an oven at 60 °C for 24 h to control the moisture. The physical characteristics of the fine and coarse aggregates (such as density, specific gravity, amount of voids, evaporable moisture contents, and WA) were recorded using the methods prescribed by ASTM C128. These parameters met all the requirements of normal mortar production, the maximum allowed limit needed by the ASTM C33 standard. The bulk density of the dried fine aggregates and specific gravity on the saturated surface dry (SSD) state were, correspondingly, 1730 kg/m^3^ and 2.70. The maximum size and fineness modulus of the fine aggregates were 4.75 mm and 2.3, respectively. The air-dried crushed granite with the highest size of 10 mm, and a specific gravity of 2.7 and 0.5% WA, was used as coarse aggregate in all prepared concrete mixes. Our systematic procedure ensured that the aggregates are devoid of organic matters such as dry muds, leaves, and other harmful components.

### 2.2. Mix Design Proportions

The proportions of the mix were calculated using the Department of the Environment (DOE 1997) method of concrete design following BS 8500-1 and 2 (2002) to classify the class of exposure related to the application of concrete to obtain the mix proportions. In the first round, the water/binder ratio was determined to be 0.56 based on the target strength of 30 MPa at 28 days and crushed coarse aggregates with a maximum size of 10 mm. However, for checking the limitation of water-to-cement ratio (w/c) based on durability considerations for exposed concrete, the approach taken did not allow a w/c ratio of more than 0.50, so the water-to-binder ratio (w/b) was fixed at 0.5. In the second round, based on the desired slump value between (30–60 mm), and the crushed coarse aggregates used with a maximum size of 10 mm, the water content was determined to be 230 kg/m^3^. Accordingly, the cement content was calculated to be 460 kg/m^3^. After that, based on the specific gravity of the used coarse aggregates (2.7), the percentage passing of the used fine aggregates on a sieve of 600 µm (40%), and water content of 230 kg/m^3^, both fine and coarse aggregates were determined to be 880 and 820 kg/m^3^, respectively. In the case of replacing cement with 50% FA, the DEO method suggested a reduction in the water content by 30 kg/m^3^. However, for the purpose of comparing the performance of the modified concrete with conventional concrete, the water amount was kept as it is. Then, FA was utilized to substitute 50% OPC by weight, keeping the same other mixing proportions as they are in the control mixture. Similarly, the amount of EMs was used to replace 10% of the mixing water. Then, various levels of WGBNPs (2, 4, 6, 8, and 10%) were added. Table 3 provides various proportions of the concrete mix design.

### 2.3. Test Setup and Procedures

**Figure 4:**
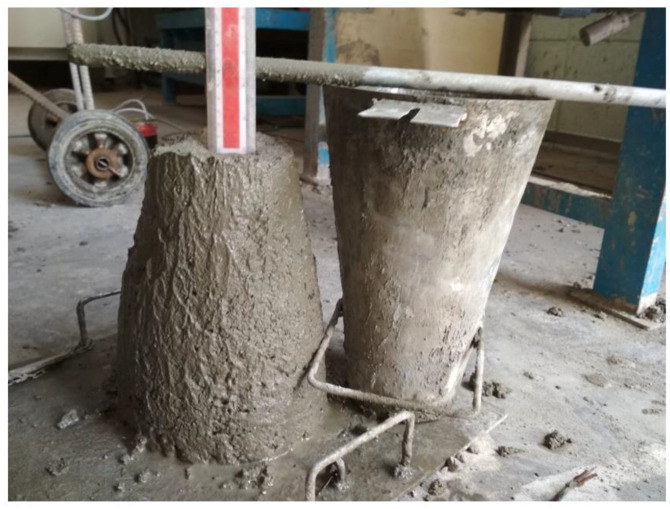
Fresh concrete’ slump test.

**Figure 5:**
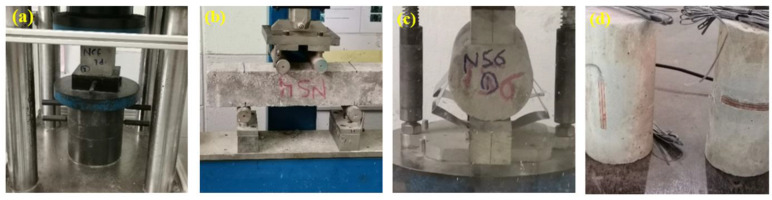
Mechanical tests of specimens’ (**a**) compressive strength, (**b**) flexural strength, (**c**) splitting tensile, and (**d**) modulus of elasticity.

The workability of the produced concrete mixes was measured by slump to assess an ease of usage of concrete in its fresh state as per ASTM C143 [53] (Figure 4). Without the addition of superplasticizer, the target slump of concrete was within the range of 60–180 mm. Three types of moulds, namely, cubes, cylinders, and prisms, were used. The samples were demoulded after 1 day and cured at ambient condition (27 °C) until tested (Figure 4c). The CS test was performed as per ASTM C109 specification [54]. The compressive strength of concrete is the most critical factor, thus the optimal percentage of WGBNP was determined on the basis of the strength results. Depending on these results, other mechanical and microstructure tests were conducted with the optimal percentage of WGBNPs. Three samples from each NC, NCF, EM, and WGBNPs optimum specimens were prepared for FS, STS, modulus of elasticity (MoE), and WA measurement. The FS of the specimens was tested in accordance with ASTM C78 [55]. The STS was evaluated as per the ASTM C496 standard [56]. The MoE was measured as per ASTM C469/C469M stipulation [57] as shown in Figure 5b–d, respectively.

The XRD, SEM, EDS, and TGA data were recorded to determine the microstructure properties of the proposed mixes. The XRD measurement and TGA/differential thermal analysis (DTA) were performed by taking a small part of the interior of the tested specimen. For the CS test, the specimen was ground to pass through a 125 nm sieve. The surface morphology of the mixes was examined by SEM. The specimens tested for strength at 7 and 28 days were taken to prepare the sample for SEM analysis, wherein it was spread onto two cellophane sheets followed by attachment onto a coin. For SEM analysis, each sample was placed in a sample holder (brass-stub-type) and dried for 5 min using ionizing radiation followed by coating with gold via a Blazers sputter coater. The SEM images were recorded at 120 kV with a magnification of 1000×. After choosing a moderately high image magnification, the morphology of the samples was recorded immediately.

## 3. Results, Analysis and Discussion

### 3.1. Fresh Properties

**Figure 6:**
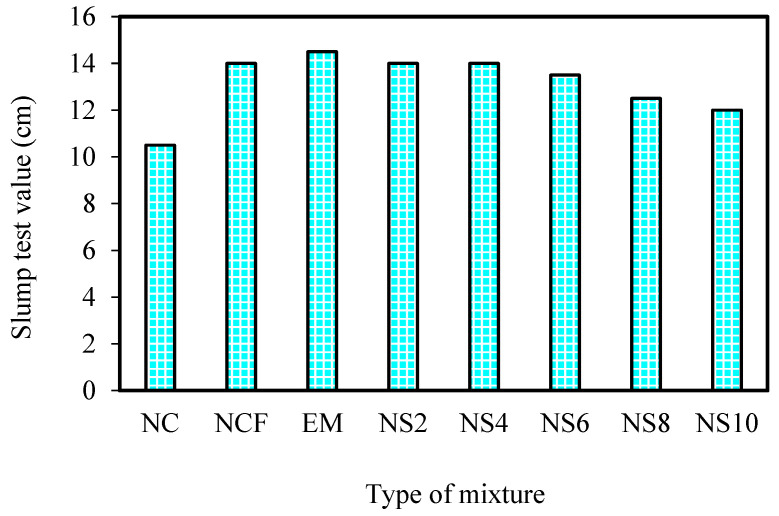
Slump test values of prepared mixtures.

Figure 6 shows the slump test results of the designed mixtures, which indicate the influence of FA in improving workability by approximately 33% with a w/c ratio 0.5. This result can be attributed to the spherical shape and smooth texture of FA particles. The slump test values were 14.5 cm with a 3.5% increase over the NCF mixture when mixing water was replaced with 10% EMs. Such findings verified the role of EMs in reducing the mixing water viscosity. Meanwhile, the addition of nanopowder resulted in a slight decrease in the workability, wherein the NS8 and NS10 samples showed a slump value reduction of approximately 17.2% and 13.8%, respectively, compared with the EM-included specimens. This observation may be attributed to the addition of nanoparticles with highly specific surface areas that required more water to become wet [38]. As opposed to spherical FA particles, the nanoglass particles with angular and irregular shapes could increase the intermolecular friction, thus limiting the workability of the specimen [29,58]. Moreover, WGBNPs tend to occupy the inter-space among mixture particles, sinking the area available for the particle dynamics, thereby requiring a high amount of water for lubricating the mixture, providing adequate workability [59].

### 3.2. Compressive Strength

**Figure 7:**
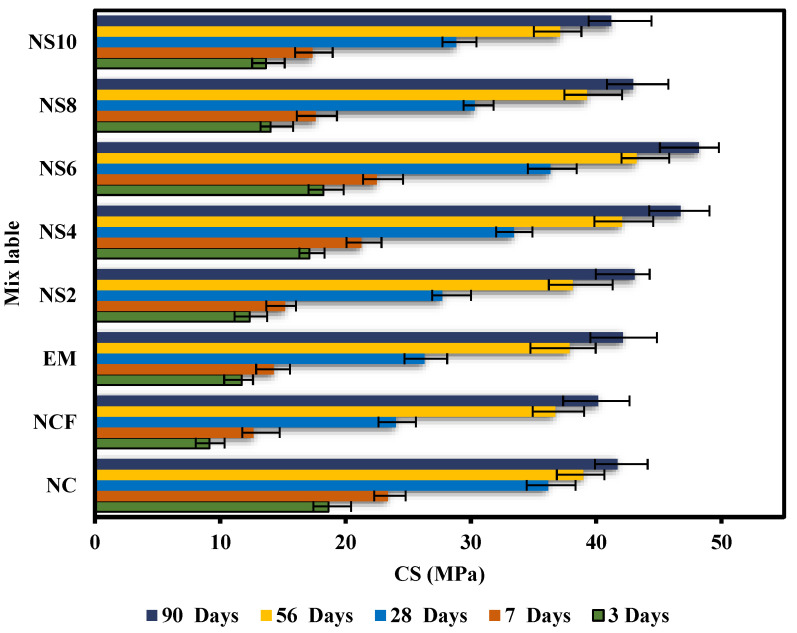
CS against curing periods of the proposed mixes.

Figure 7 shows the CS of the designed specimens after 3, 7, 28, 56, and 90 days. The NCF specimen showed a sharp reduction in compressive strength compared with the normal concrete, especially at 3, 7, and 38 days, where the reduction rates were 51.0, 45.9, and 33.6%, respectively. This reduction was due to the low cement content and weak pozzolanic nature of the FA particles. However, with the progression of the curing period, the pozzolanic reaction rate of FA was gradually increased, thus improving the strength of high-volume FA-based concrete significantly. For the concrete samples made with EMs, the replacement of 10% of the water with EMs was found to increase the strength at all ages. At 3, 7, 28, 56, and 90 days, the CSs of the EM-added concretes were 11.71, 14.26, 26.31, 37.86, and 42.14 MPa, respectively. The results indicate that the addition of EMs in the concrete with 50% FA as cement replacement increased the strength by 28.1% at 3 days. At 7 and 28 days, the strength increase was 12.7% and 9.5%, respectively. Conversely, at 56 and 90 days, the rate of strength increment declined to 4.9% and 3.1%, respectively. Several studies have reported similar increases in the strength of concretes (5–30%) prepared with different EM percentages [60,61,62,63,64,65,66,67,68,69].

With regard to the mechanisms that underlay the EMs’ enhancement of concrete properties, previous studies claimed that the addition of EMs can reduce the surface tension of the mixed water, allowing it to create tinier holes when assed to concrete; this condition contributes to the increase in strength values [43,70]. The results of this study are consistent with the one reported by Huseien et al. [71], wherein the inclusion of 10%EMs as mixing water was shown to improve strength by 42.5, 25, and 14.5% at 3, 7, and 28 curing days, respectively. Another study [72] showed that a reduction in the mixing water surface tension can produce better workability and improve the strength properties of the concrete instead of introducing extra mixing water. In general, the integration of EMs significantly boosts strength at early ages, but the rate increment reduces with the progression of the curing age. Conversely, the effect of 10% EMs inclusion as mixing water replacement and different percentages of WGBNPs as nanoadditives was more robust in terms of strength improvement. The results show that the addition of 6% WGBNPs can produce higher CS at all ages than other values. Meanwhile, the strength of the specimens made with 4% of WGBNP exhibited satisfactory performance at all curing ages. The increment rates of NS6 were 99.6, 77.8, and 51.31%, while those of NS4 were 87.3, 68.1, and 39.1% at 3, 7, and 28 days, respectively. At 56 and 90 days of age, the CS of NS4 and NS6 surpassed the values of NC, with the values 42.06 and 46.74 MPa for NS4, 43.22 and 48.19 MPa for NS6 and 38.96, and 41.71 MPa for NC at 56 and 90 days, respectively. The improvement in the strength of all samples made with WGBNPs may be mainly because of: (1) the pozzolanic reaction of WGBNPs and (2) the effects of WGBNPs in pore filling and enhancing specimens’ density. Nevertheless, at 3, 7, and 28 days, the NS2, NS8, and NS10 specimens showed lower strength than NC, which may be due to the nonideal filling action. Nonetheless, the strength of NS2 and NS8 was either equivalent to or greater than that of the strength at 56 and 90 days. However, the strength value of NS10 was reduced to 2.0 and 2.2% at 56 and 90 days, respectively, compared with the specimen made with EMs. At early ages, the high surface area of WGBNPs facilitated the strength enhancement of NS10, making it relatively superior. However, its performance remained lower than NS4 and NS6 samples because of the decreasing interior spaces needed to generate hydration reaction products. In brief, with the increase in nanoparticles contents, the adjacent space among the nanoparticles was lowered, inhibiting the creation of Ca(OH)_2_ crystals.

**Figure 8:**
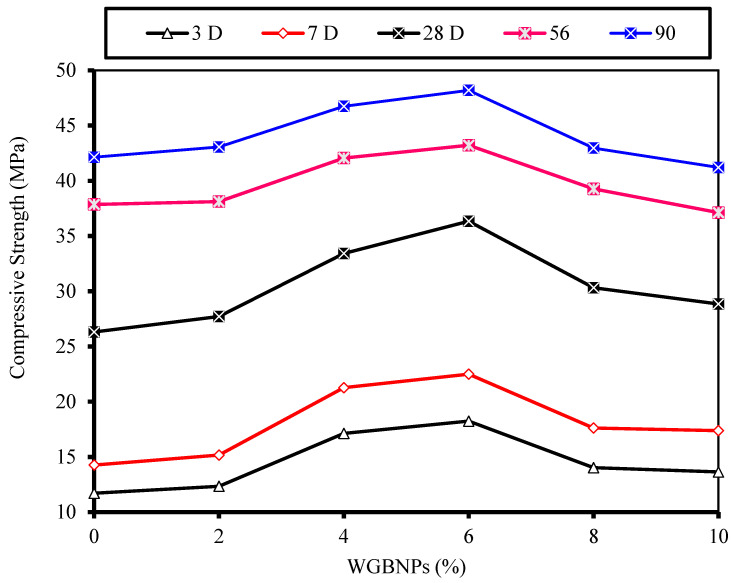
Influence of WGBNPs on strength, where 0% refers to the strength of the EM specimen.

Figure 8 depicts the WGBNPs’ content-dependent variation of strength development. The optimal percentage occurred closer to 6% and in the range of 4 to 6%. Still taking 4% as the optimum, the percentage achieved a satisfactory performance to generate more information about the role of WBGNPs for other mechanical performance parameters and microstructures.

### 3.3. Flexural Strength

**Figure 9:**
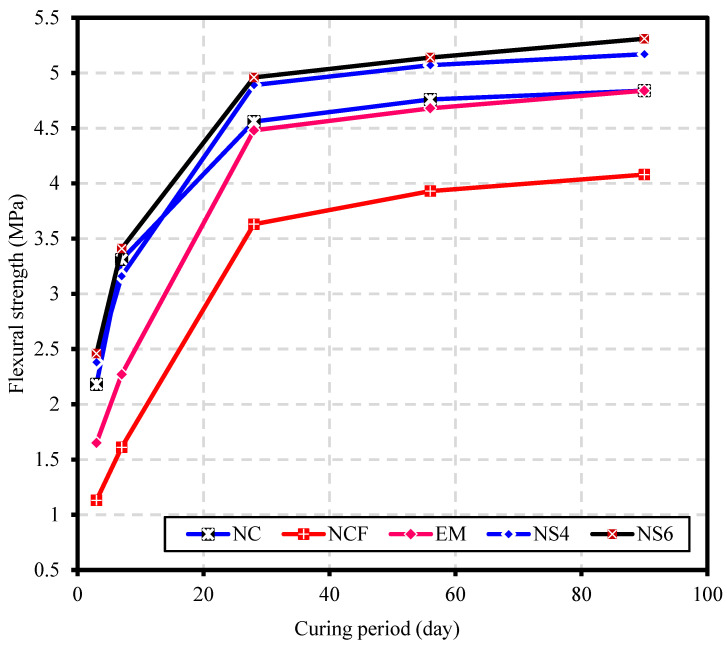
Flexural strength against curing period of various studied specimens.

Figure 9 shows the flexural strength of the NC, NCF, EM, NS4, and NS6 mixes at 3, 7, 28, 56, and 90 days. At the age of 3 days, the flexural strength of NC and NCF was 2.18 and 1.13 MPa, respectively, while at 7 days, the FSs of the NC and NCF specimens were 3.31 and 1.61 MPa, respectively. The reduction rate of NCF specimens was compared with the control sample (NC). The reduction at earlier ages was higher, which was 48.2 and 51.4 at 3 and 7 days, respectively. The reduction in flexural strength associated with replacing 50% of OPC by FA was attributed to a lack of hydrates resulting from cement hydration and the high porous microstructure of the NCF specimen. It became known that the pozzolanic reaction of silicates embedded in FA with Ca(OH)_2_, generated by cement hydration, leads to the formation of a C-S-H gel, which is the major contributor to gaining strength. However, when the proportion of fly ash in concrete increases, the cement content becomes insufficient to bond the particle aggregates enough, and the calcium hydroxide produced by cement hydration becomes inadequate to activate a large portion of FA content for the pozzolanic reaction. This leaves considerable amount of FA content unreacted in the mix, which increases pore size and overall porosity of the matrix, resulting in a drop in FS. In the case of the EM specimens, the enhancement rate of flexural strength was 46.0, 41.0, and 23.4% at 3, 7, and 28 days, respectively, as compared with NCF. This result can be ascribed to the role of EMs in reducing the mixing water surface tension, enabling some water to escape into the connected pores and accelerate the hydration reaction.

The observed slight reduction in pH could influence the kinetics of hydration in the early stages, where a study mentioned that the solubility of Ca(OH)_2_ increases with a slight reduction in the pH value of the solution, which could have a positive effect on the cement hydration process [73]. Additionally, the inclusion in EM and FA in the OPC network was responsible for reducing the heat of hydration, providing an appropriate environment to improve the OH^–^ contents and pH, thus leading to the formation of more gel products. However, this contrasts with the studies that confirm the Si and Al solubility increase with increased solution alkalinity. Therefore, it is difficult to confirm the effect of a slight decrease in the pH value on the strength indicators of high-volume FA-based concrete at early ages. In accordance with the findings of Rahman et al. [74] and Andrew et al. [67], the addition of EMs contributed approximately 6% and 4% improvement to the FS of concrete, respectively. The authors ascribed the improvement of the FS to the role of EMs in producing denser microstructures, better connection bonds between particles, and stronger interfacial transition zones.

In the presence of WGBNPs, the findings revealed that the flexural strength of both specimens NS4 and NS6 exceeded that of NC. The FS of NS4 and NS6 were 2.38 and 2.46 MPa at 3 days, as well as 3.16 and 3.41 MPa at 7 days, respectively. Likewise, at 28 days, both samples of NS4 and NS6 achieved an FS of 4.89 and 4.96 MPa, respectively. The increased rates of FS of NS6 were 117.7, 111.8, and 36.6%, while the increment rates of the NS4 specimens were 110.6, 96.3, and 34.7% at 3, 7, and 28 days, respectively.

The increase in flexural strength at early ages can be mainly ascribed to the existence of fine WGBNPs particles that served as seeds, providing extra nucleation sites and expediting the hydration process in the mixes [75]. Furthermore, the highly specific areas of WGBNPs increase their pozzolanic reactivity, forcing them to react faster compared with FA content. The dissolution of amorphous silica in WGBNP produced H_2_SiO_4,_ which reacted with Ca^2+^, forming C-S-H gel [17]. The formation of a high number of hydrates may strengthen bridging bonds, especially at interfacial transition zones, improving the flexural strength. The good distribution of WGBNPs within the matrix is also considered to have a positive role in terms of the homogeneity of the microstructure of the concrete. This causes the hydration products to spread uniformly throughout the matrix, eliminating any defects caused by heterogeneity. The presence of WGBNPs with good distribution was shown to improve pore structure, reduce the percentage of harmful pores, and refine the microcracks. At curing ages of 56 and 90 days, the increased values in the FS of NS6 were 30.8 and 30.1%, whereas these values for NS4 were 29.0 and 26.7%, respectively. It might be said that at later ages, as the fly ash particle activity increases, the dramatic fall in flexural strength is overcome in samples lacking WGBNPs, which may explain the decrease in the increment rates in flexural strength at 56 and 90 days.

### 3.4. Splitting Tensile Strength

**Figure 10:**
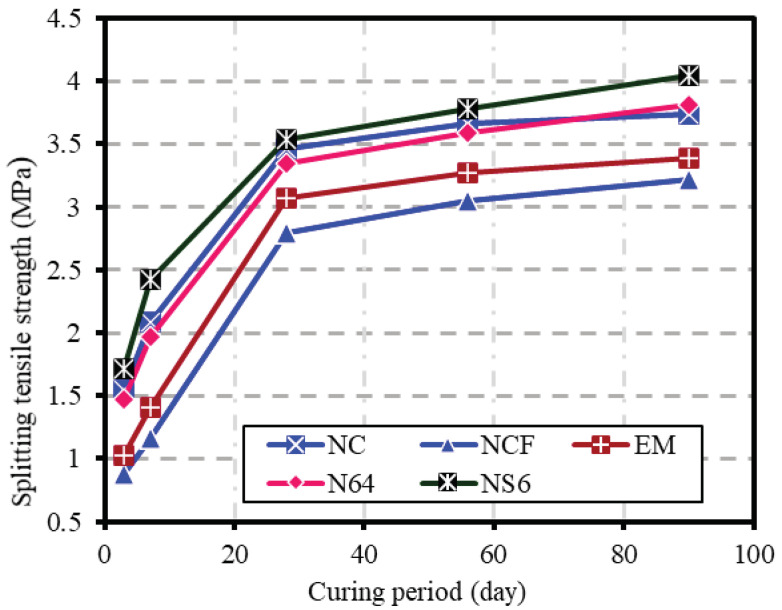
Variation of splitting tensile strength against curing periods.

Figure 10 displays the average splitting tensile strength obtained for the NC, NCF, EM, NS4, and NS6 specimens at 3, 7, 28, 56, and 28 days. The tensile strengths of NC were 1.54, 2.08, 3.46, 3.66, and 3.74 MPa at 3, 7, 28, 56, and 90 days of curing ages, respectively. Meanwhile, the STSs of the NCF specimens were 0.88, 1.16, 2.8, 3.05, and 3.22 MPa at 3, 7, 28, 56, and 90 days, respectively. Similar to compressive strength and FSs, the reduction in splitting tensile strength due to OPC replacement by 50% FA was generally remarkable. The reductions in strength of the NCF specimens compared with that of NC were 42.9, 44.2, 19.1, 16.7, and 13.9% corresponding to 3, 7, 28, 56, and 90 days. The observed high reduction in strength at early ages was due to the slow pozzolanic reaction of FA and deficiency of OPC content needed to bond aggregate particles. At 56 and 90 days of curing period, the gap reduction in split strength between NCF and NC was notably reduced. The higher development rate of the split strength of NCF at late ages was closely related to accelerating the pozzolanic activity of fly ash particles.

In the presence of EMs, the tensile strength was significantly improved. The increment rates of EM compared with NCF were 17.0, 21.6, and 9.6% at 3, 7, and 28 days, respectively. This may be due to the role of EMs in lowering the surface tension of mixed water, allowing water to diffuse into the linked pores and speeding up the hydration process and thus allowing more packing, especially at early ages [49]. With the progress of curing age, the improvement rate decreases gradually. The enhancement rate of split strength resulting from replacing 10% of mixing water with EMs was 7.2% and 5.3% at 56 and 90 days, respectively. This can be attributed to the improvement in the split strength in the comparison samples (NCF) due to the pozzolanic reaction of FA. During the later ages, the C_2_S of cement continues to react, raising Ca(OH)_2_ concentration, forcing FA to dissolve and react to form further hydrates. The secondary hydration products can fill the pore voids, which are beneficial to the split strength of concrete and contribute to the densification of the transition zone at the interface between the aggregate and the paste, resulting in faster growth of strength at later ages. However, in the case of EM specimens, the enclosing of hydration products around unreacted FA particles at early ages and the increased compactness of samples could limit the ability of some amount of unreacted FA to react at later ages.

Similar to CS and FS, specimens containing 4 and 6% WGBNPs showed higher performance regarding split tensile strength. The NS6, which contains 50% FA as cement replacement and 10% EMs as mixing water replacement, recorded higher split strength than the control sample of normal concrete, where the increments were 94.3, 108.6, and 26.4% at 3, 7, and 28 days, respectively. Likewise, the enhancement rate in NS4 was also notable, where it was 67.0, 69.8, and 19.6% at 3, 7, and 28 days, respectively. The main reason for such strength development was the strong pozzolanic reaction of WGBNPs and the decrease in pore size distribution. Similar results show the role of fine glass powder in the development of strength indices to their strong pozzolanic traits and high surface-area-dependent reactivity [35,76]. Elaqra et al. [35] used fine glass powders in different mixes with and without bottom ash and observed an increase in STS at about all curing ages. The observed improvement in STS was due to the shrinking of pore sizes originating from pozzolanic reaction and formation of compact hydrate gels [35]. Furthermore, WGBNPs might have acted as seeds and fillers within the pores and small cracks, providing excess nucleation sites, thus improving the network compactness and reducing the porosity, positively influencing the strength characteristics [45]. At later ages, the NS6 specimen remained superior in split strength performance, even to normal concrete. Compared with the NCF specimens, the rates of improvement in tensile strength were approximately 23.9 and 25.5% at 56 and 90 days, respectively. The specimen of NS4 also revealed a similar performance to that in normal concrete (NC). The persistence of superior split strength performance at later ages is attributed to the accelerating role of FA in improving strength performance.

### 3.5. Modulus of Elasticity

**Figure 11:**
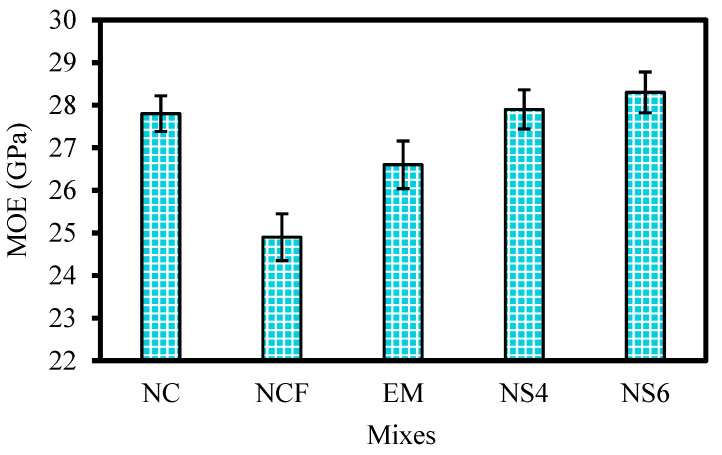
Modulus of elasticity of the designed mixes.

Figure 11 shows the modulus of elasticity values of the designed mixtures at 28 days. The modulus values of the mixtures ranged from 24.9 to 28.3 GPa. The results demonstrate that the modulus of the NCF specimen exhibited a low modulus. The modulus of the NCF specimen was 24.9 GPa, with a reduction of 10.4% compared with the control sample (normal concrete). This reduction may be due to the retarded pozzolanic process of FA [77,78,79,80]. Replacing mixing water with 10% EMs increased MOE from 24.9 GPa to 26.6 GPa. However, the modulus of the EM specimens remained lower than that of the NC sample by approximately 4.3%. This reduction is attributed to the high effect of replacing OPC with FA. By contrast, the modulus of the EM specimen increased by 6.8% compared with that of NCF. The increase in modulus is due to the inclusion of EMs, consistent with the results of Liu et al. [81] and Reddy et al. [82]. Similarly, the modulus is influenced positively by the inclusion of WGBNPs. The modulus at 28 days of curing was 27.9 and 28.3 GPa for the NS4 and NS6 specimens, respectively. Notably, the NS6 specimen demonstrated significantly better performance with respect to the modulus compared with all the other samples, including NC. The increment rate in the modulus of NS6 was 1.8% compared with the normal concrete (NC), while that of NS4 was not notable (i.e., 0.4%). To determine the influence of the integrated inclusion of EMs and WGBNPs on the modulus, comparing the NS4 and NS6 samples with the NCF sample is preferable. Evidently, the integrated inclusion of 4 and 6% WGBNPs as nanoadditives, and 10% EMs as mixing water replacement, to a binary mixture (OPC and FA) resulted in a 12% and 13.7% increase in moduli, respectively. In terms of the role of glass powders, several studies [24,83,84] were performed using glass as the replacement for cement. The results of all these studies show an insignificant increase in the MoE, particularly in early ages. However, it seems that by improving the glass fineness at nanoscale, the pore filling and nucleation sites can be increased [17]. Additionally, the nanoparticles’ addition can reduce Ca(OH)_2_ crystal growth, thus reducing the possibility of the formation of weak areas within the matrix, improving the modulus of elasticity [85,86].

### 3.6. Water Absorption

**Figure 12:**
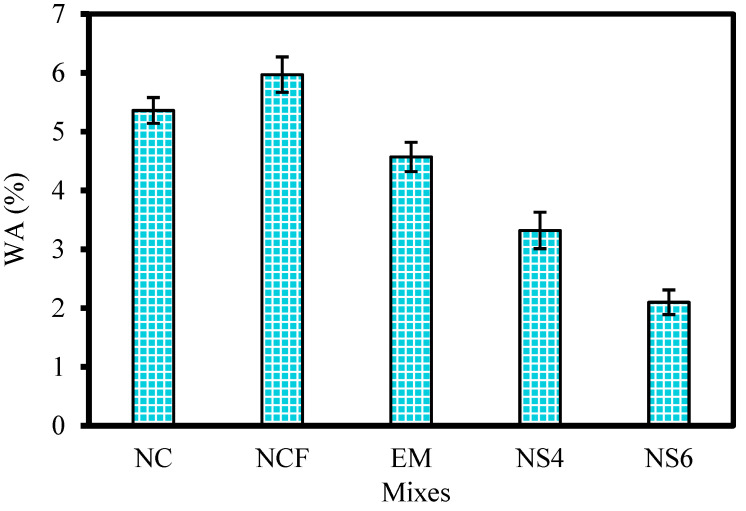
Water absorption rate at 28 days.

Figure 12 shows the WA at 28 days of all designed mixes. The NCF specimen exhibited a higher water absorption percentage (5.97%) than that of the control sample (NC), with an increment rate of 11.4%. The observed increase may be ascribed to the slow rate of pozzolanic reaction of FA, leaving a significant amount of capillary pores that enabled diffusion of water into the concrete matrix. When mixing water was replaced with 10% EMs, the water absorption decreased to 4.57%, with reduction rates of 14.7 and 23.5% compared with the NC and NCF specimens, respectively. As mentioned previously, the role of EMs is lowering the mixing water surface tension, controlling the pore size and distribution, and reducing the confined water within the pores, as well as reducing the permeability of specimens containing EMs. The results of the current study are compatible with those of Huseien et al. [43]. In the case of WGBNPs’ inclusion, the NS6 specimen expectedly showed the lowest WA rate. With the inclusion of 10 EMs as mixing water replacement, and 4% and 6% WGBNPs to the binary mixture prepared with OPC and FA, the water absorption percentages were 3.32% and 2.1% for NS4 and NS6, respectively. The reduction rates in water absorption of NS4 and NS6 compared with those of the NCF specimen were 44.4 and 64.8%, respectively. Patel et al. [87] and Parghi et al. [88] found that the incorporation of glass nanoparticles into concrete can reduce permeability, and consequently water absorption. The low water surface tension resulting from the EMs solution and the strong pozzolanic reaction of WGBNPs might have impacted positively the hydration mechanism, leading to the creation of denser gels that lowered the ratio of pores in the specimen’s matrix prepared with 10% EMs and WGBNPs compared with the NC and NCF mixes. Furthermore, the homogeneous distribution of WGBNPs into the concrete’s network produced more dense and homogeneous microstructures, controlling the capillary and cracks network, thus reducing water absorption.

### 3.7. Microstructure Analyasis

**Figure 13:**
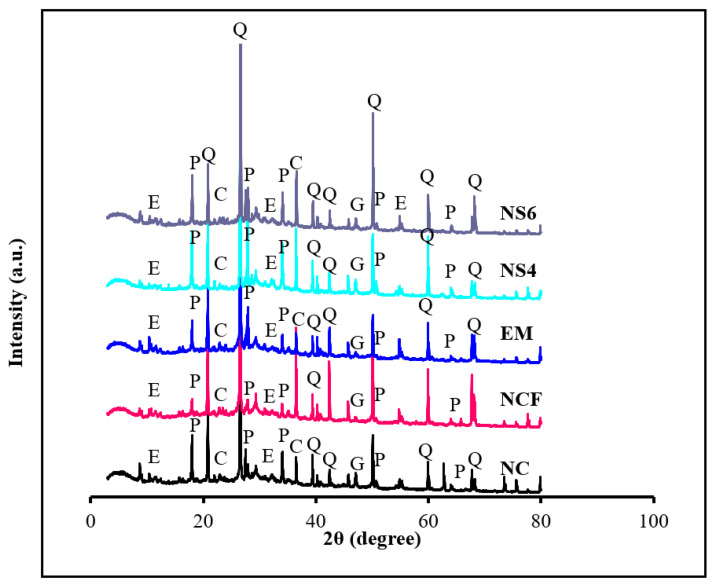
XRD patterns of the designed mixes at 28 days (Q: quartz; C: calcite; E: ettringite; P: portlandite; G: gypsum).

Figure 13 illustrates the powder XRD profiles of the NC, NCF, EM, NS4, and NS6 mixes at 28 days. The addition of a high content of FA (50%) as a cement substitute was found to have a negative effect on the hydration reaction, restricting the overall Ca(OH)_2_ and CaCO_3_ gels’ formulation. With the increase in FA contents, more nonreacted silica was produced, as displayed by XRD peaks. The intense diffraction peaks at 20.1, 39.8, 50, and 60.4° corresponded to the existence of quartz (SiO_2_), portlandite (Ca(OH)_2_), calcite (CaCO_3_), ettringite (Ca_6_Al_2_(SO_4_)_3_(OH)_12_.26H_2_O), and gypsum (CaSiO_4_) phases originated from OPC and FA blending. The quartz peak intensity was enhanced slightly for the mix made with 50% of FA as OPC substitute due to the presence of more nonreactive quartz than NC, resulting in a decrease in CS from 36.16 to 24.02 MPa at 28 days. The strong reaction of amorphous SiO_2_ and Al_2_O_3_ in FA with highly crystalline C_3_S, C_2_S, and C_3_A in OPC was responsible for such products’ formation. Conversely, the NC specimen displayed portlandite and calcite peaks of about 18–65°. Nonetheless, by substituting OPC with 50% of FA, the portlandite peaks around 18–50° appear weaker, which illustrates the low strength of NCF compared with NC. Unlike with the inclusion of 10% EMs as mixing water replacement and 4 and 6% WGBNPs as nanoadditive, the intensity of the quartz peaks were lowered at the cost of the peak intensity of portlandite because more silica was dissolved, thus formulating the C–(A)–S–H gel via the hydration reaction. With the replacement of mixing water by EMs, a slight increase in the peaks of Ca(OH)_2_ was noted compared with those in the mixture with only 50% of FA. This indicted the hydration reaction relatively improved, raising compressive strength by 9.5% at 28 days, respectively. This result can be ascribed to the role of EMs in accelerating the hydration process. However, the peaks of Ca(OH)_2_ increase slightly when WGBNPs were added. This could be attributed to the behavior of WGBNPs as seeds, providing further nucleation sites to precipitate further hydration products. Likewise, an increase in the peak intensities of CaCO_3_ at 29.32° and 50.22° enhanced strength from 26.31 to 33.42 and 36.34 MPa.

The quartz peaks exhibited no uniform trend, and this finding may be related to the possibility of grinding aggregates and the difference in their percentage in the tested powder, leading to the disruption of the uniform trend of quartz peaks. Generally, the substitution of cement by FA produced a considerable decrease in the intensity of Ca(OH_2_) and C-S-H peaks, which may be ascribed to the deficiency of calcium contents in the proposed mixes, which justifies the reduction in strength in NCF concrete. A relative increase in Ca(OH_2_) peaks was also observed when mixing water was replaced with 10% EMs, and a similar increase was recorded in calcite intensity peaks in both NS4 and NS6 concrete, which illustrates the increase in strength parameters.

**Figure 14:**
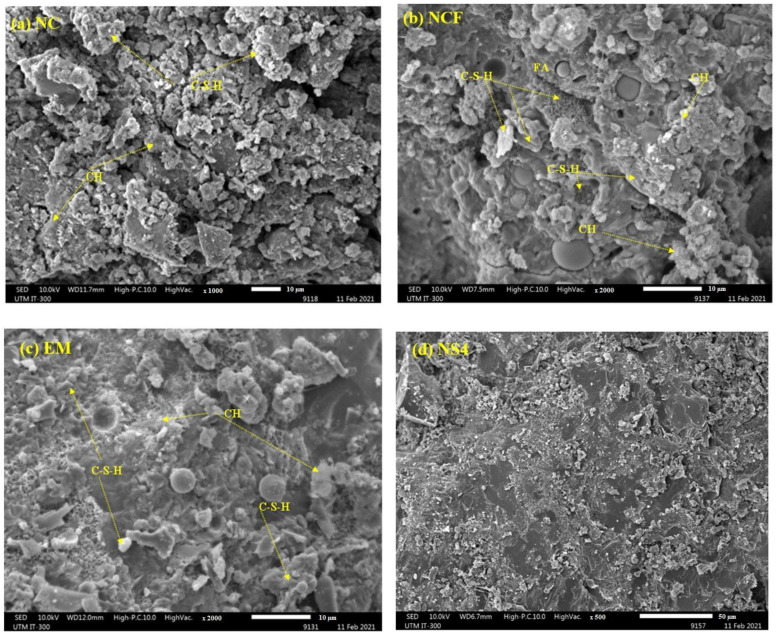

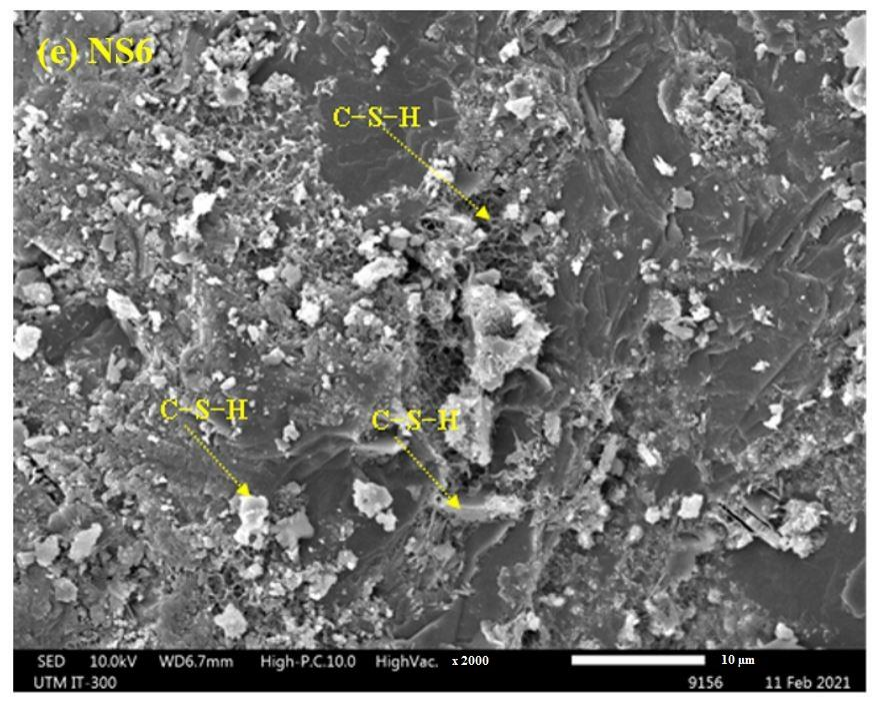
(**a**–**e**) SEM micrograph of the designed concrete.

Figure 14a–e shows the SEM micrograph of the prepared concrete specimens at 28 days. The less dense and less homogeneous microstructure of NC was remarkable, thus recording a high water absorption of 5.36% at 28 days. As seen in Figure 14b, some hydration products were identified in the FA surface, indicating the hydration of FA particles. Nonetheless, some unhydrated FA particles with even surfaces were also seen. According to the repost of Lam et al. [89], the reaction extent of FA after 28 days in the concrete made with 55% FA and a water-to-binder ratio of 0.5 was 9.82%, indicating that a substantial percentage of FA had not yet begun to hydrate at this age. So, the low strength values are attributable to the low production of a C-S-H gels (or C-A-S-H gels) with a lower calcium-to-silicon (Ca:Si) ratio from the secondary hydration process of FA. However, the NCF concrete showed a denser microstructure than that of NC due to the pores filling, providing extra nucleation sites in the transition regions and pores. In addition, it was predicted that the hydration process and pozzolanic reaction could form more C–(A)–S–H gels, increasing the microstructure packing densities at late ages. With the inclusion of 10% EMs as mixing water replacement, the hydration/pozzolanic process of the concrete samples was enhanced and presented less unreacted FA particles and pores. The enhanced hydration reaction raises the alkalinity of the solution, catalyzing the solubility of silica–aluminum content in FA, activating FA particles to react faster. Consequently, more (C–(A)–S–H) gels were formulated than the specimen, made only using cement and FA, mainly accounting for the enhancement of the strength parameters of EM concrete. However, some amount of FA content remained unreacted, as shown in Figure 14c, which illustrates the relative reduction in compressive strength of the EM specimen compared with NC. In mixtures containing 10% EMs as a replacement for mixing water and 4% and 6% WGBNPs, comparatively dense microstructures were seen. As illustrated in Figure 14d,e, FA particles were wrapped by hydration-reaction-related secondary products, and these mixes also contained higher hydration products, indicating that a significant portion of FA started to hydrate. This refers to the inclusion of WGBNPs enhancing the pozzolanic activity of the FA nanosphere, leading to the formation of further C–(A)–S–H gel, increasing packing of the microstructures, thus improving strength indexes at 28 days. Through SEM observations, it is possible to observe that the presence of EMs and WGBNPs introduced more dense and more homogeneous microstructures, which reflects positively on mechanical performance.

**Figure 15:**
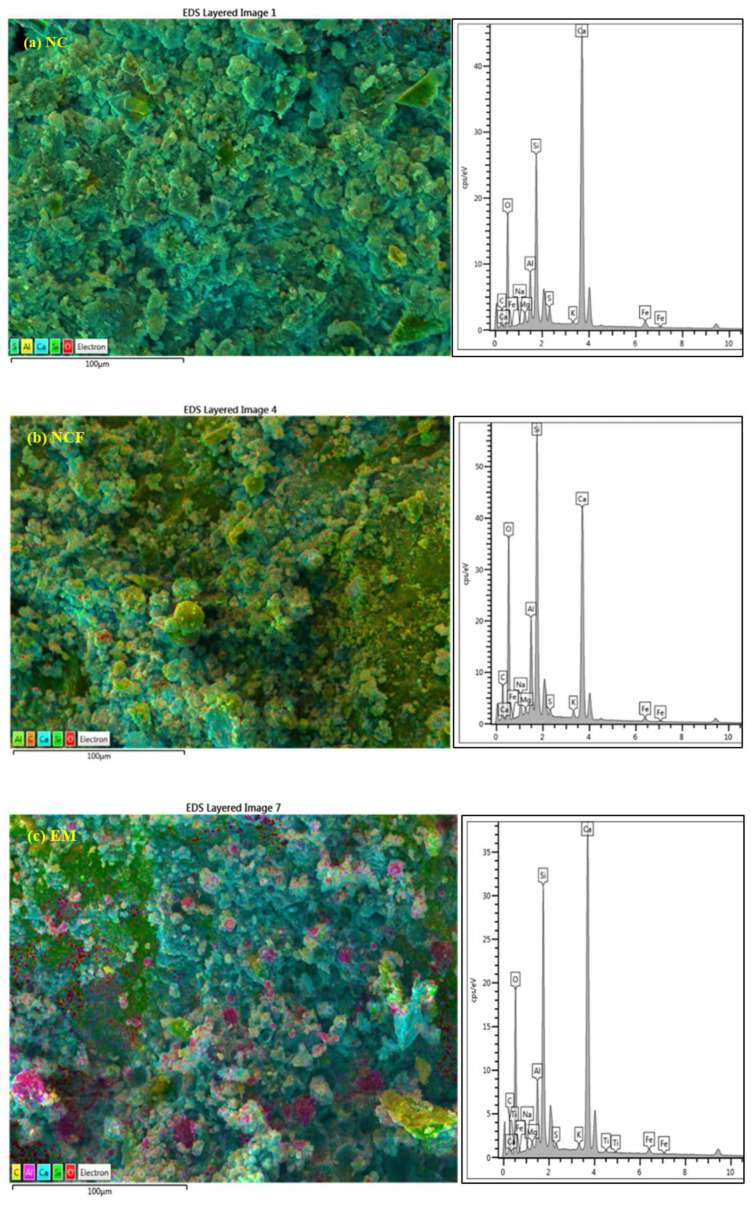

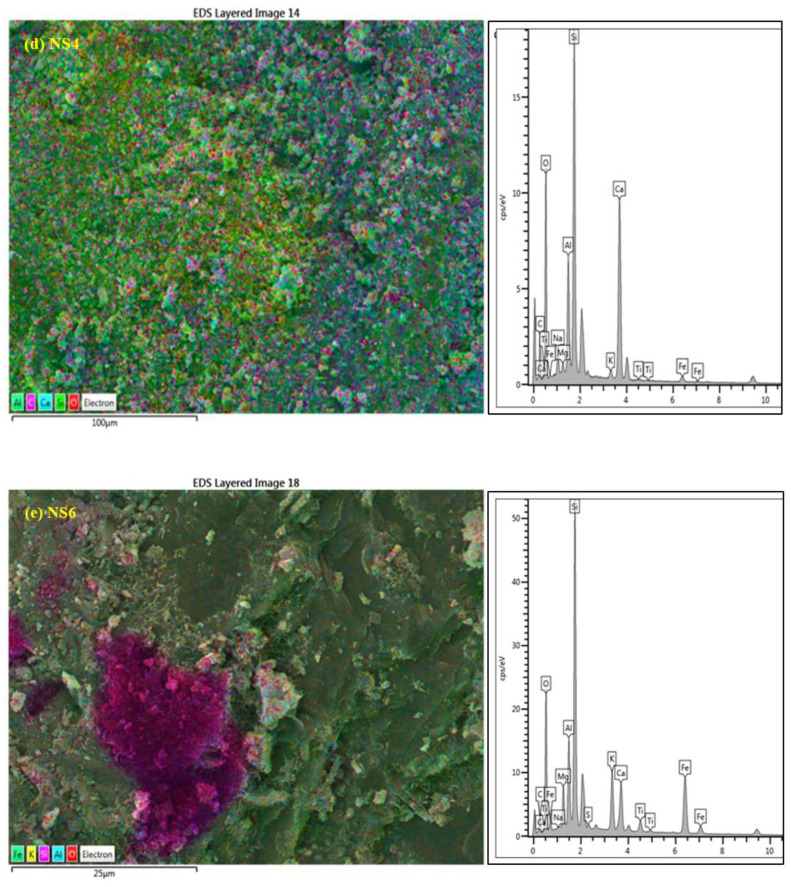
(**a**–**e**) EDS spectra of the designed concrete.

Figure 15a–e shows the element mapping of NC, NCF, EM, NS4, and NS6 after 28 days of curing. The EDS spectra indicated a high calcium-to-silicon (Ca/Si) proportion of 3.2 in the NC specimen. However, the Ca content was reduced, while the Si and Al contents were increased when OPC was substituted with 50% FA, with Ca/Si being 1.31 and the calcium-to-aluminum ratio (Ca/Al) being 3.7. However, the concrete prepared with 50% FA as cement replacement and 10% EMs as mixing water replacement presented a high Ca/Si of 2.7 and a Ca/Al of 8.1, which were attributed to high Ca(OH)_2_ content due to the acceleration of the OPC reaction. The low percentage of Al content in the EM specimen compared with that in NCF can be attributed to Al being consumed in the formation of C–S–H and C–(A)–S–H chains. Furthermore, the silicon-to-aluminum ratio (Si/Al) was increased from 2.8 to 3.6 with the inclusion of EMs. The observed increase in compressive strength with an increase in Ca(OH_2_) led to the formation of more gels compared with that of the concrete prepared only with 50% OPC and 50% FA as cement replacement. With the inclusion of WGBNPs, the Si, Al, and sodium (Na) contents increased, whereas Ca percentage decreased. The Ca/Si and Ca/Al also decreased from 2.7 to 0.96 and 0.25 and from 8.1 to 2.73 and 0.89 with the addition of 4 and 6% of WGBNPs, respectively. The reduction in Ca/Si and Ca/Al was ascribed to consumption of Ca content in the formation of complicated chains of C–S–H and C–(A)–S–H gels. Conversely, the inclusion of 10% EMs and WGBNPs increased the Si/Al from 2.8 to 3.5, increasing the compressive strength from 24.02 to 36.34 MPa, respectively. The increase in compressive strength with an increase in Si/Al was ascribed to the consumption of Al in further C–(A)–S–H chains. In general, a decrease in Ca/Si and Ca/Al indicates a consumption of Ca(OH)_2_ in the pozzolanic reaction and formation of further hydration products, which is reflected in an increase in the strength of specimens with WGBNP. Low Si concentrations (low Si/Al) could be due to the formation of a poly(sialate) structure, in which silicon is replaced by Al. The trend of compressive strength was observed to increase as the Si/Al ratio increased, while Ca/Si decreased. This finding is compatible with the findings of the researchers in [90,91,92,93], who discovered that the inclusion of silica-rich materials, such as silica, leads to the development of C–S–H/C–A–S–H with a lower Ca/Si and Ca/Al. Clearly, the incorporation of Si–Al-rich materials leads to the creation of C–S–H/C–(A)–S–H with distinct and low Ca/Si and Ca/Al ratios. Furthermore, the studies also reported that decreased Si/Al ratios refer to consuming Si^+4^ in the formation of a Si–O–Si structure, which is one of most stable silicate chains.

**Figure 16:**
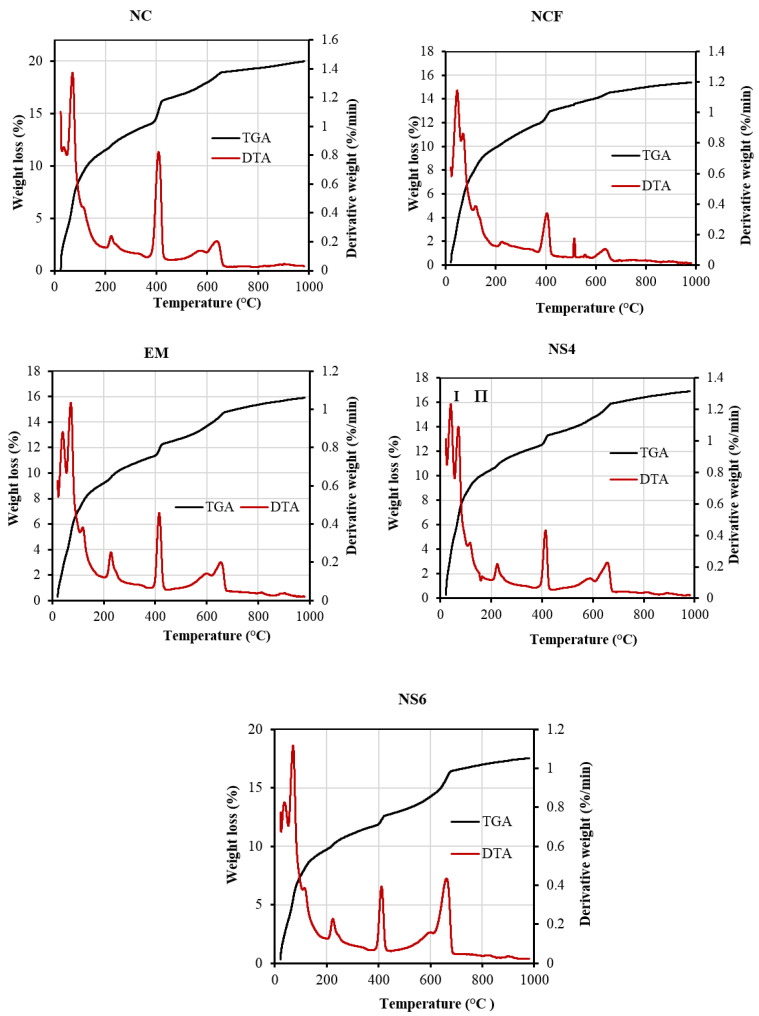
TGA and DTA results of the designed specimens at 28 days.

Figure 16 depicts the thermal stability of the HVFA-modified concrete samples that included EMs and WGBNP at temperatures ranging from 20 to 1000 °C. The TGA and DTA were performed to evaluate the weightloss percentage of the proposed modified mixes. The amount of Ca(OH)_2_ and CSH was obtained using the corresponding Equations (1) and (2):

Ca(OH)_2_ (%) = WL Ca(OH)_2_ (%) × [MW Ca(OH)_2_/MW H], (1)

where WL(Ca(OH)_2_) is the weightloss ascribed to Ca(OH)_2_ dehydration, MW(Ca(OH)_2_) is the molecular weight of Ca(OH)_2_ (74 g.mol^−1^), and MW(H) is the molecular weight of water (18 g.mol^−1^).

C–S–H gel (%) = Total LOI − LOI Ca(OH)_2_ − LOI CC (2)

Table 4 presents the TGA results of the mixes produced using FA, EMs, and WGBNPs at 7 and 28 days. The TGA and DTA data indicated that the percentage of C–S–H gel in the specimen made with 50% FA as OPC substitution was lower (9.94%) than the OPC specimen (16.25%) at 28 days. In addition, the results show a low amount of Ca(OH)_2_ in the specimen made with 50% FA (4.81%) compared with the OPC specimen (8.21%). The lower stability of concrete made with 50% FA and the small quantity of C–S–H gel and Ca(OH)_2_ clearly indicated a negative effect of a low amount of Ca in FA in the formulation of dense gels, leading to the less dense microstructures of the NCF specimen. The low amount of hydration products caused a drop in CS from 36.16 MPa to 24.02 MPa at 28 days with the substitution of OPC by 50% FA. For specimens with EMs, replacing mixing water with 10% EMs led to an increase in C–S–H gel amount from 9.94 to 13.25%, enhancing compressive strength from 24.02 to 26.31 MPa at 28 days, respectively. Similarly, the hydration process was enhanced with the inclusion of WGBNPs, producing more dense gel. Essentially, the addition of 4 and 6% WGBNPs increased the C–S–H gels from 13.25 to 14.17 and 14.92%, increasing compressive strength from 26.31 to 33.42 and 36.34 MPa, respectively. In contrast, the inclusion of 4 and 6% of WGBNPs reduced the Ca(OH)_2_ from 4.93 to 4.52 and 3.69%, respectively, indicating the role of WGBNPs in the enhancement of the pozzolanic reaction. The better stability of the FA-based concrete with 4 and 6% WGBNPs can be attributed to the formation of the high amount of hydrate gels with a low quantity of Ca(OH)_2_ [94].

## 4. Conclusions

Various contents of WGBNPs and EMs were integrated with a series of high-volume FA-based concretes to boost early strength properties. In these proposed mixes, OPC was replaced with 50% FA by weight of binder, and mixing water was replaced with 10% EMs by weight of water. Then, WGBNPs were added in the proportions of 2, 4, 6, 8, and 10% weight of the binder. The finding indicated a significant and positive effect of EM and WGBNP inclusion on the overall performance and strength properties of the proposed concrete mixes at all studied curing ages. The following conclusions can be drawn based on the main findings:

- The workability of prepared concrete is significantly influenced by FA, EM, and WGBNP content. The value of the slump was increased with the replacement of 50%OPC and inclusion of 10% EMs as mixing water replacement, while decreasing with the addition of WGBNPs.
- The strength values were enhanced due to the inclusion of 10% EMs as mixing water replacement and 4 and 6% WGBNPs at early ages.
- The replacement of 50% OPC with FA enhanced the WA value, while the inclusion of 10% EMs as mixing water replacement and 4 and 6% WGBNPs led to a significant decrease in WA (%).
- The incorporation of 10% EMs as mixing water replacement and 4 and 6% of WGBNPs boosted the pozzolanic reaction process, thus increasing the formation of hydrate gels and less unreacted cement/FA.
- The synergy of EMs and WGBNP was found to make the microstructures denser and more homogeneous than the binary mixtures prepared with OPC and FA only, which explained the reason for their higher mechanical performance at early ages.
- Microstructure results (XRD, SEM, EDs, TGA, and DTA) show that the inclusion of EMs and WGBNPs in concrete increased the content of hydration products, leading to high stability and better mechanical performance.
- In short, high-volume FA-based concrete containing local waste materials (glass bottles and effective microorganisms) can be beneficial to achieve sustainable construction materials by reducing landfill problems, saving energy consumption, and lowering the demand for natural resources for concrete production, thus diminishing environmental pollution.
- Although the pH value of effective microorganism solution is lower, which is not a favorable condition for corrosion, effective microorganisms also contain 97.5% rhodium, which has good resistance of corrosion. So, concrete containing effective microorganism solution should be assessed by detailed corrosion investigation.

## Figures and Tables

**Table 1:** Physical and chemical properties of raw materials.

Elements	Materials		
	OPC	FA	WGBNPs
SiO_2_	17.60	57.20	69.14
Al_2_O_3_	4.53	28.81	13.86
Fe_2_O_3_	3.35	3.67	0.24
CaO	67.84	5.16	3.16
MgO	2.18	1.48	0.68
K_2_O	0.27	0.94	0.01
SO_3_	-	0.10	4.08
Na_2_O	-	0.08	0.01
Loss on ignition, LOI	1.73	0.12	0.16
Blaine fineness-BET (cm^2^/g)	3995	-	-
Average diameter (µm)	16.4	10	0.12
Specific gravity	3.15	2.20	1.02
Color	Dark Grey	Grey	Light grey
Specific surface area (m^2^/g)	-	18.2	206

**Table 2:** Obtained values of viscosity coefficient, pH, and surface tension of EMs–water mix.

Solution	Viscosity, mPas	pH	Surface Tension, mN/m
Water	0.95	6.7	66
EMs	1.44	3.45	35.6
90% water + 10% EMs	1.05	6.3	54.5

**Table 3:** Mix design proportions.

No.	Mix	Binder (kg/m^3^)			Solution (kg/m^3^)		Aggregate (kg/m^3^)	
		OPC	FA	WGBNPs	Water	EMs	Fine	Coarse
1	NC	460	0	0	230	0	880	820
2	NCF	230	230	0	230	0	880	820
3	EM	230	230	0	207	23	880	820
4	NS2	230	230	9.2	207	23	880	820
5	NS4	230	230	18.4	207	23	880	820
6	NS6	230	230	27.6	207	23	880	820
7	NS8	230	230	36.8	207	23	880	820
8	NS10	230	230	46	207	23	880	820

NC: Traditional concrete (control sample); NCF: Concrete containing 50% of FA as OPC replacement; EM: Concrete containing 10% of EMs as water replaceement; NS2, 4, 6, 8 and 10: Concretes containing 2, 4, 6, 8 and 10% of WGBNPs, respectively.

**Table 4:** The hydration products percentages calculated by TGA results at 28 days.

Parameter	NC	NCF	EM	NS4	NS6
C–S–H gel (%)	16.25	9.94	13.25	14.17	14.92
Ca(OH_2_) (%)	8.21	4.81	4.93	4.52	3.69

## Data Availability

Not applicable.
